# Comparison of efficacy between triamcinolone acetonide and triamcinolone hexacetonide for intraarticular therapy in juvenile idiopathic arthritis: a retrospective analysis

**DOI:** 10.1186/s41927-022-00249-z

**Published:** 2022-03-31

**Authors:** Angela Chun, Lutfiyya N. Muhammad, Deirdre De Ranieri

**Affiliations:** 1 Division of Rheumatology, Texas Children’s Hospital, Houston, TX USA; 2 Department of Pediatrics, Baylor College of Medicine, Houston, TX USA; 3 Division of Biostatistics, Department of Preventive Medicine, Northwestern University Feinberg School of Medicine, Chicago, IL USA; 4 Division of Rheumatology, Ann & Robert H Lurie Children’s Hospital, Chicago, IL USA; 5 Department of Pediatrics, Northwestern University Feinberg School of Medicine, 225 East Chicago Avenue, Box 50, Chicago, IL 60611-2605 USA

**Keywords:** Injections, Intraarticular, Corticosteroids, Juvenile idiopathic arthritis, Triamcinolone acetonide, Triamcinolone hexacetonide

## Abstract

**Background:**

There are many FDA-approved corticosteroid preparations available for intra-articular injection, however triamcinolone hexacetonide is not one of them. It was the intraarticular drug of choice among pediatric rheumatologists up until approximately a decade ago, when production of this medication ceased. It can be obtained in the United States and Canada via importation from Europe, but it is not FDA-approved at this time. We wish to compare the duration of remission of intraarticular triamcinolone hexacetonide (TH) with that of triamcinolone acetonide (TA) in children with juvenile idiopathic arthritis (JIA) and demonstrate its safety in this population.

**Methods:**

This retrospective chart review included 39 patients with JIA who received intraarticular corticosteroid injections (IACIs) from September 2018 to September 2019. These patients were reviewed and their life-time injections with either TH (41 joints) or TA (124 joints) was noted through May 30, 2021. Patients with concomitant systemic therapy initiation were excluded. The primary outcome was time to relapse. Relapse was defined by the presence of arthritis on physical examination by an attending rheumatologist. Kaplan–Meier curves and a log-rank test were constructed to compare the probability of time to relapse between IACI injections. Additionally, mixed effects cox regression models were constructed to account for multiple injections per participant.

**Results:**

Kaplan–Meier estimator of median relapse time in months was higher for TH. Based on the log-rank test, TA joints had a higher probability of experiencing a relapse during the study time (*p* value < 0.001). The hazard of time to relapse was reduced when comparing TH to TA in both unadjusted and adjusted mixed effects cox regression models [unadjusted hazard ratio (95% confidence interval): 0.184 (0.089, 0.381); adjusted hazard ratio (95% confidence interval): 0.189 (0.092, 0.386)].

**Conclusions:**

TH has longer duration of action than TA and is associated with less systemic side effects. It should be considered the drug of choice for intraarticular corticosteroid injections in children with JIA.

## Background

Intraarticular corticosteroid injections (IACIs) have long been considered a safe and effective treatment option in the management of patients with juvenile idiopathic arthritis (JIA) [1–4]. IACIs are used to achieve rapid resolution of arthritis, with the goals of providing pain relief and preventing joint damage [1]. The use of IACIs is associated with significantly less systemic toxicity than taking corticosteroids orally, intramuscularly, or intravenously [5, 6]. Intraarticular corticosteroids are often used as initial therapy in patients with Oligoarticular JIA and as adjunctive therapy in patients with other subtypes of JIA who may be flaring in a limited number of joints or either initiating or transitioning between different systemic therapies [1, 7]. In JIA, several studies have reported long term benefits of IACIs, including long term reduction in pain and inflammation [1, 8], stabilization and/or prevention of limb length discrepancy [3, 4], as well as resolution of joint pannus [9].

There are several different intraarticular corticosteroid formulations available. Lower solubility agents have been shown to have slower absorption and longer duration of action, leading to higher efficacy [1, 10]. Triamcinolone acetonide (TA) and triamcinolone hexacetonide (TH) are the most commonly used formulations of injectable corticosteroids in North America and Europe [11]. TA and TH differ only in the presence of one side chain, but their efficacy is markedly different. Several studies have shown TH to be superior to TA in duration of clinical remission in patients with JIA [1, 10–13]. This could potentially be due to its slower absorption and release [10]. Additionally, TH has been shown to specifically reduce synovial T cell lymphocytes with associated decrease in inflammatory cytokines [14, 15].

The commercial production of TH (labelled as Aristospan) in the United States was halted in 2015. Due to the lack of availability of Aristospan, pediatric rheumatologists returned to using other steroid formulations, such as TA, for intraarticular injections. Another brand of TH (labelled as Lederspan) became available to patients who have failed TA injections through the Personal Importation Policy (PIP) as set forth by the Food and Drug Administration (FDA). The PIP allows the importation of certain medications for which there are no currently FDA-approved acceptable alternatives in the United States and the patient has failed the similar FDA-approved available medication. At Ann & Robert H Lurie Children’s Hospital, we were granted access to this medication and have been using it for over 2 years in patients who have failed TA, as evidenced by a flare in arthritis.

In order to compare the efficacy of TH to the current standard of TA in maintaining clinical remission for participants with JIA, we performed a retrospective study to compare time to relapse between TH and TA. Our primary outcome was time to relapse based on patient report and physician’s clinical exam. Additionally, since the knees were the most commonly injected joints in children with JIA, we performed a subgroup analysis to assess time to relapse for participants that received IACIs into the knee joint only.

## Methods

This protocol was reviewed and approved by the Institutional Review Board at Ann & Robert H Lurie Children’s Hospital (IRB 2019-2951). In this retrospective chart review, EMR CPT codes identified patients, diagnosed with JIA according to the International League of Associations for Rheumatology (ILAR) criteria [16], who received an IACI with either TA or TH at a single tertiary center between September 1, 2018 and September 1, 2019. These patients were reviewed, and their life-time injections through May 30, 2021, were included in the analysis.

Patients with systemic JIA and undifferentiated arthritis were excluded. To minimize confounding bias, patients who had received an IACI within 3 months prior to the injection or those who had started a new systemic medication or were transitioning between systemic medications within 3 months of the injection were excluded. Medical records were reviewed for patient demographics and clinical course.

Lederspan (a brand of TH) is a German medication that is not currently FDA approved, so it was obtained via the Personal Importation Policy (PIP), which allows a patient to import a foreign non-FDA approved medication if they have failed the available FDA-approved medication (TA). All of the patients in this study had failed TA and were thus eligible for TH based on this policy. The medication was obtained from Germany via the Canadian pharmaceutical company, Medexus Pharma.

Ultrasound guided IACIs were performed by the same provider using standard techniques, either with topical anesthesia (using a J-tip, which is a sterile, single use, subcutaneous needle-less injection device) or under sedation with an anesthesiologist. Standardization of dosages for TA were as follows: large joints (knees, hips, shoulders) received 60–80 mg depending on the size of the patient; medium joints (ankles, wrists, elbow) received 40 mg; and small joints (fingers, toes) received 4–8 mg. Standardized dosages for TH were as follows: large joints (knees, hips, shoulders) received 40 mg; medium joints (ankles, elbow) received 30 mg, the wrist received 20 mg; and small joints (fingers, toes) received 4–6 mg. After IACI, patients who had knees and hips injected were advised to minimize their activity for a period of at least 24 h, and up to 48 h, as this has been shown in adults to improve outcome in larger joints [2, 17–19].

Relapse was defined by the presence of active arthritis in the joint per an attending pediatric rheumatologist’s physical exam. Active arthritis was defined as swelling within the joint, and if no appreciable swelling was present, other signs and symptoms suggestive of arthritis such as limitation in range of motion, pain with movement of the joint, and inflammatory type symptoms such as morning stiffness, were used to assess activity.

Demographics and patient characteristics were compared between IACI types using the Mann–Whitney U test for non-normal continuous variables and Fisher’s exact test for categorical variables. Time to relapse in months was the primary outcome in our analyses. Kaplan–Meier curves and log-rank tests with all joints and knees only were constructed to compare the probability of time to relapse between IACI groups. Participants were censored if they did not experience a relapse during the study duration. Unadjusted and adjusted mixed effects cox regression models were constructed to account for multiple injections per participant. IACI type (TA vs. TH) was the primary predictor in the models. The adjusted model for all joint locations included age, sex, ethnicity, JIA diagnosis categories, and joint injection location. The covariates in the adjusted model for knee-only joint injections were age, sex, ethnicity, and JIA diagnosis categories. Hazard ratios (HRs) and 95% confidence intervals (CI) of the HRs summarized the findings from the mixed effects cox regression models. Due to several participants receiving both TA and TH during the study period, we conducted a sensitivity analysis of the unadjusted and adjusted mixed effects cox regression models with participants that received TA and TH removed from the analyses.

## Results

Demographics and participant characteristics are listed in Table 1.

**Table 1:** Demographics and participant characteristics by IAC injection type

	TA N = 34 with 124 injections	TH N = 14 with 41 injections	*p* value
Relapse time in months (median [IQR])	3.00 [2.00, 6.00]	11.00 [5.00, 18.00]	< 0.001*
Age in years (median [IQR])	4.88 [2.92, 10.65]	4.83 [3.02, 7.67]	0.901
JIA disease duration in years (median [IQR])	0.46 [0.19, 2.66]	1.17 [0.44, 4.33]	0.179
Female (%)	27 (79.4)	10 (71.4)	0.708
Ethnicity (%)			0.160
White	27 (79.4)	13 (92.9)	
Black	4 (11.8)	0 (0.0)	
Hispanic	3 (8.8)	0 (0.0)	
Unknown	0 (0.0)	1 (7.1)	
JIA diagnosis (%)			0.461
Oligo	24 (70.6)	12 (85.7)	
Poly	8 (23.5)	1 (7.1)	
Psoriatic	2 (5.9)	1 (7.1)	
Injections by joint locations (%)			0.051
Knee	62 (50.0)	24 (58.5)	
Ankle	31 (25.0)	5 (12.2)	
Wrist	14 (11.3)	1 (2.4)	
Elbow	2 (1.6)	1 (2.4)	
Fingers/toes	14 (11.3)	9 (22.0)	
Hip	1 (0.8)	0 (0.0)	
Mid-foot	0 (0.0)	1 (2.4)	

Total sample size is 39 participants with 165 injections. Nine of the 39 participants received both IAC injection types. *p* values from Mann–Whitney U tests and Fisher’s Exact tests are listed are for the comparison between IAC injection types

*IQR* interquartile range

*Denotes *p* value < 0.05

A total of 39 JIA patients with 165 IACIs are included in this study. Nine of the 39 participants received both injections. There were 34 participants that received 124 TA injections, and 14 participants received 41 TH injections. Median relapse time in months for the TH group was significantly longer than the TA group (median TA relapse time = 3 and median TH relapse time = 11, Mann–Whitney U test *p* value < 0.001). The median age in both groups were approximately the same (TA median age = 4.88 and TH median age = 4.83, Mann–Whitney U test *p* value = 0.901). Median JIA disease duration in years at time of injection was not statistically different between groups (TA median JIA disease duration = 0.46 and TH median JIA disease duration = 1.17, Mann–Whitney U *p* value = 0.179). In both groups, there were more females than males. There were no statistical differences between groups for ethnicity, JIA diagnosis subtype, and injections by joint locations. For both TA and TH, the knee was the most frequently injected joint.

**Fig. 1:**
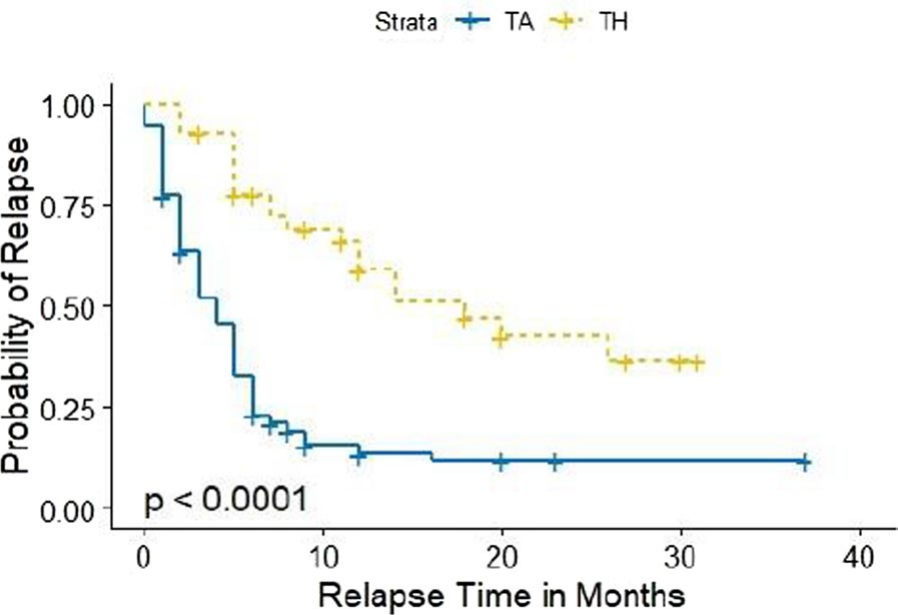
Kaplan Meier curves by IAC injection type for all joints. Censored values are denoted by a plus sign. The *p* value in the figure indicates the *p* value from log-rank test that compares the injections

**Fig. 2:**
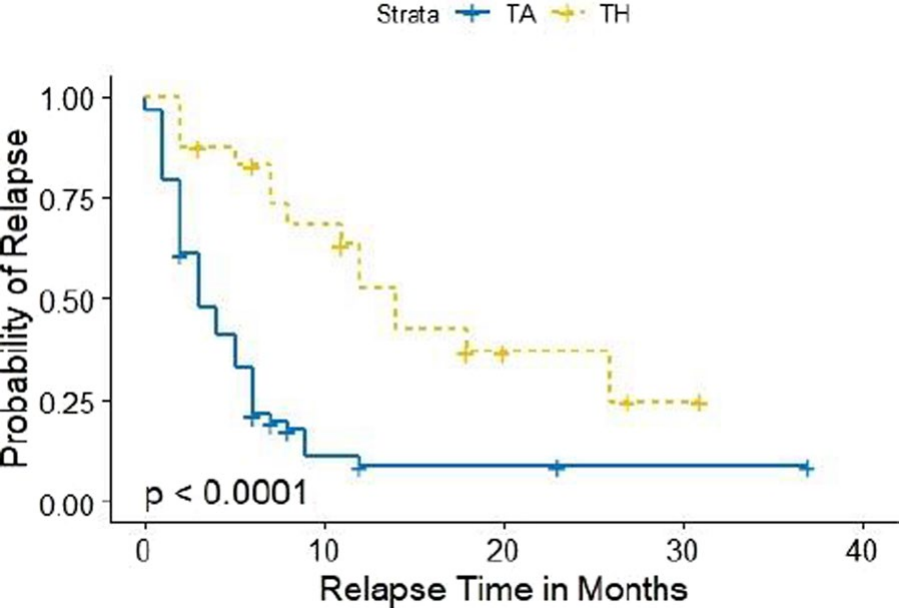
Kaplan Meier curves by IAC injection type for knee only. Censored values are denoted by a plus sign. *p* value listed in the figure represents the log-rank test *p* value that compares the injection types

Figures 1 and 2 illustrates the Kaplan–Meier plots for all injections and knee only injections respectively.

The Kaplan–Meier estimator of the median relapse time in months was higher for TH in comparison to TA (TH median = 18 and TA median = 4). Based on the log-rank test, there was a difference between TA and TH in the probability of experiencing a relapse during the study time (*p* value < 0.001). For the knee only analysis, the Kaplan–Meier estimator of the median relapse time for the TH group was 14 months while the Kaplan–Meier estimator of the median relapse time was 3 months for those that received TA. There was a difference between TA and TH knee injections in the probability of experiencing a relapse during the study period (log-rank *p* value < 0.001).

The adjusted mixed effects cox regression model results for all injections and knee only injections are in Tables 2 and 3 respectively.

**Table 2:** Hazard ratios from mixed effects cox regression models that included injections in all joints

	Hazards ratio (95% CI)	*p* value
Injection: TH versus TA	0.189 (0.092, 0.386)	< 0.001*
Age in years	0.996 (0.893, 1.112)	0.950
Females versus males	0.555 (0.177, 1.743)	0.310
JIA diagnosis: oligo versus other	0.346 (0.127, 0.943)	0.038*
Joint: knee versus other	1.413 (0.918, 2.174)	0.120
Ethnicity: white versus other	0.439 (0.116, 1.659)	0.220

Sample size is 39 participants with 165 injections. Nine participants received both IAC injection types. The mixed effects cox model included age, sex, ethnicity, diagnosis (oligo vs. other (poly, psoriatic, and ERA)), and joints (knee vs. other (ankle, wrist, elbow, finger/toes, shoulder, hip, SI, and TMJ)) as covariates

*Denotes *p* value < 0.05

**Table 3:** Hazard ratios from mixed effects cox regression models with participants that received IAC injections in the knee only

	Hazards ratio (95% CI)	*p* value
Injection: TH versus TA	0.131 (0.052, 0.322)	< 0.001*
Age in years	0.946 (0.831, 1.077)	0.400
Females versus males	0.407 (0.104, 1.593)	0.200
JIA diagnosis: oligo versus other	0.208 (0.055, 0.782)	0.020*
Ethnicity: white versus other	0.536 (0.010, 2.882)	0.470

Sample size is 21 participants with 86 injections, and nine participants received both IAC injection types. The mixed effects cox model included age, sex, ethnicity, and JIA diagnosis (oligo vs. other (poly, psoriatic, and ERA)) as covariates

*Denotes *p* value < 0.05

The hazard of time to relapse was reduced when comparing the TH group to TA group in both the unadjusted and adjusted mixed effects cox regression models (unadjusted HR = 0.184, 95% CI (0.089, 0.381), *p* value < 0.001; adjusted HR = 0.189, 95% CI (0.092, 0.386), *p* value < 0.001). The reduced hazard implies that time to relapse was longer for the TH group relative to the TA group. In our sensitivity analysis that excluded data from the nine participants that received both TH and TA, we found that the hazard of time to relapse was significantly reduced when comparing the TH group to TA group in both the unadjusted and adjusted mixed effects cox regression models (unadjusted HR = 0.039, 95% CI (0.006, 0.242), *p* value < 0.001; adjusted HR = 0.075, 95% CI (0.015, 0.368), *p* value = 0.001).

Similarly, participants in the knee injections only analysis that received TH had a longer relapse time than those that received TA. The estimated unadjusted and adjusted HRs that compared TH to TA for those that received knee injections only were both statistically significant (unadjusted HR = 0.109, 95% CI (0.043, 0.278), *p* value < 0.001; adjusted HR = 0.131, 95% CI (0.052, 0.322), *p* value < 0.001). Based on our sensitivity analysis results of the knee injections only data with the participants that received both TH and TA removed, the TH group had a longer relapse time relative to the TA group in the unadjusted and adjusted models, but the HR in the adjusted model was marginally statistically significant (unadjusted HR = 0.102, 95% CI (0.017, 0.582), *p* value < 0.010; adjusted HR = 0.315, 95% CI (0.099, 1.004), *p* value = 0.051).

## Discussion

Intraarticular corticosteroid injections (IACIs) are often used as first-line therapy in patients with Oligoarticular JIA, in whom a limited number of joints are affected. IACIs are also used as adjunctive therapy in other subtypes of JIA to expeditiously control inflammation and decrease pain while awaiting systemic medications to take effect. IACIs have the benefit of quickly decreasing inflammation, thus minimizing the risk of morbidity associated with JIA, such as cartilage destruction, muscle wasting, and leg length discrepancies [1–4, 14]. Acquired leg length discrepancies in children with JIA are thought to result from stimulation of the growth plate due to the inherent hyperemia in the region secondary to synovial inflammation [4]. Intraarticular corticosteroids have been shown to effectively reduce synovial T lymphocytes and downregulate certain pro-inflammatory cytokines, including TNF-a, IL-1b, extranuclear HMGB-1, ICAM-1, and VEGF [15].

Historically, TH has been shown in several studies to have a longer duration of action and a superior side effect profile compared to other intraarticular corticosteroid formulations [10–13, 20]. TH was superior, even at lower doses, with effects lasting up to 24 months [10, 11].

Furthermore, it has been shown to be an effective therapy for inflammatory arthritis in all subtypes of JIA [21]. Aristospan, a formulation of triamcinolone hexacetonide (TH), was the preferred corticosteroid for intraarticular injection until it became unavailable in the United States almost a decade ago. Since that time, pediatric rheumatologists have been using Kenalog, a brand of triamcinolone acetonide (TA). However, there have been very few contemporary studies comparing the efficacy of the two.

The results from our study support the past comparative studies in pediatric rheumatology that have suggested superiority of TH compared to TA across all subtypes of JIA. TA dosing was approximately 50% higher than customary dosing (there is no established dosing regimen for TA), similar to prior studies comparing these two drugs, The median time to flare in joints treated with TH was 11 months in our study, which is similar to the mean time to flare reported by Eberhard et al. of 10.14 ± 0.49 months in the group injected with TH [13]. Lepore et al. reported a mean duration of remission of 13.9 months in knees injected with TH for patients with oligoarticular JIA [22]. This slightly longer duration of remission may be secondary to the isolation of knee injections, with previous studies suggesting IACIs were most effective in this joint [8, 13, 21]. Similar to us, both Eberhard et al. and Lepore et al. defined remission as a complete disappearance of clinical signs of inflammation. Other studies have reported much longer durations of remission. Zulian et al. compared the efficacy of TH and TA in oligoarticular JIA in a prospective study, with almost double the response rate with TH at 24 months [10]. Subsequently, Zulian et al. compared TA at twice the dose of TH in children with symmetric arthritis in a prospective double-blinded study, with similar findings [11]. However, the scale to assess for arthritis was different, allowing for a nominal degree of arthritis. In a retrospective study, Marti et al. reported a longer median duration of remission of 23.1 months for patients who underwent IACI with either TH or TA, but patients were often started on concomitant medications at time of injection [8].

The median time to flare in joints treated with TA in our study was 3 months, which is shorter than the mean time to flare of 7.75 ± 0.49 months reported by Eberhard et al. However, it should be noted that Eberhard et al. used 80 mg for the knee and 60 mg for the elbow, ankle, and wrist, which is higher than what was used in this study. Some studies suggest that higher doses of TA are needed to be effective, and while our dosing of TA was approximately 50% higher than customary dosing, the dosing in this study was 100% higher [10, 11, 13]. Additionally, every patient in our study who received TH had previously failed IACI with TA, suggesting a more refractory disease group and the potential for a more robust response if initially treated with TH.

Side effects of IACI are usually mild and temporary, with discomfort at the injection site being the most common [23]. Other reported side effects include mood and sleep alteration, appetite changes, menstrual irregularities, weight gain, and Cushingoid appearance, especially in young patients or in those getting multiple injections [24, 25]. Skin hypopigmentation and subcutaneous atrophy from leakage of the steroid along the needle track can also be seen, but these changes usually resolve with time [1, 25]. In our cohort, the use of TH for IACI was associated with more post-procedural discomfort, but fewer systemic side effects, likely due to increased intraarticular residence and less systemic absorption [25]. Compared to those who received TA, those who received TH had less post-procedural emotional lability, appetite changes and weight gain, flushing and malaise.

Limitations of this study include the small sample size and the retrospective nature of chart reviews. The sample size was limited by the availability of the medication, as the cost was not covered by insurance. Furthermore, there was often parental hesitance to use a medication that was not FDA-approved. Interestingly, more males than females received TH compared to TA, and the reason for this is unclear, i.e., if the medication was offered to more male patients, or if the parents of male patients were more likely to want to try it (as opposed to starting systemic therapy). We know that in this age group, in general, there is a female predominance, so that will be an interesting variable to evaluate going forward.

The majority of the patients in this study had Oligoarticular JIA (both persistent and extended), as is expected. Most of the patients that we treat who have either psoriatic or polyarticular JIA or have uveitis receive systemic therapy initially, and those patients that received concurrent joint injections were not included. The patients that were included in the study either received joint injections as initial management or if they flared on systemic therapy in which case they were only included if therapy did not change before or after they received joint injections. However, by excluding patients who concomitantly started or changed systemic therapy, we may have introduced some selection bias, as there was no standardization for stepwise escalation of therapy. This could suggest that patients with worse disease may have been quickly started on concomitant therapies and excluded from the study. However, we would argue that since all patients were required to have previously failed TA injections to qualify for TH injections, this may suggest that they had more severe or refractory disease at baseline.

A future goal is to conduct a prospective study evaluating the duration of remission of TH versus TA, when TH is used in steroid-naïve joints as opposed to being used as a second-line agent for intraarticular corticosteroid injections.

In conclusion, we confirm the finding that TH is superior to TA in terms of time to flare and associated side effects, and we advocate for increased availability of this product for our patients. At the time of this writing, Medexus Pharma is the sole pharmaceutical company with rights to sell this drug in the United States. They are currently importing Trispan from France, which is the same chemical formulation as Lederspan which was imported from Germany.

Literature to support the superiority of TH over TA could expedite the FDA approval of this medication and render it less cumbersome for pediatric rheumatologists to obtain.

## Data Availability

The datasets used and/or analysed during the current study available from the corresponding author on reasonable request.

## Abbreviations

JIA: Juvenile idiopathic arthritis
TA: Triamcinolone acetonide
TH: Triamcinolone hexacetonide
FDA: Food & Drug Administration
IACI: Intraarticular corticosteroid injection
ILAR: International League Against Rheumatism
EMR: Electronic medical record
CPT: Current procedural terminology
